# Inhibition survey with phenolic compounds against the δ- and η-class carbonic anhydrases from the marine diatom *thalassiosira weissflogii* and protozoan *Plasmodium falciparum*

**DOI:** 10.1080/14756366.2019.1706089

**Published:** 2019-12-19

**Authors:** Siham A. Alissa, Hanan A. Alghulikah, Zeid A. ALOthman, Sameh M. Osman, Sonia Del Prete, Clemente Capasso, Alessio Nocentini, Claudiu T. Supuran

**Affiliations:** a Chemistry Department, College of Science, Princess Nourah bint Abdulrahman University, Riyadh, Saudi Arabia; b Chemistry Department, College of Science, King Saud University, Riyadh, Saudi Arabia; c Istituto di Bioscienze e Biorisorse, CNR, Napoli, Italy; d NEUROFARBA Department, Sezione di Scienze Farmaceutiche e Nutraceutiche, Sezione di Scienze Farmaceutiche e Nutraceutiche, Università degli Studi di Firenze, Sesto Fiorentino, Firenze, Italy

**Keywords:** Diatom, protozoan, metalloenzyme, δ-/η-class, inhibition

## Abstract

The inhibition of δ- and η-class carbonic anhydrases (CAs; EC 4.2.1.1) was poorly investigated so far. Only one δ-CA, TweCA from the diatom *Thalassiosira weissflogii,* and one η-CA, *PfCA,* from *Plasmodium falciparum*, have been cloned and characterised to date. To enrich δ- and η-CAs inhibition profiles, a panel of 22 phenols was investigated for TweCA and *PfCA* inhibition. Some derivatives showed effective, sub-micromolar inhibition of TweCA (K_I_s 0.81–65.4 µM) and *PfCA* (K_I_s 0.62–78.7 µM). A subset of compounds demonstrated a significant selectivity for the target CAs over the human physiologically relevant ones. This study promotes the identification of new potent and selective inhibitors of TweCA and *PfCA*, which could be considered as leads for finding molecular probes in the study of carbon fixation processes (in which TweCA and orthologue enzymes are involved) or drug candidates in the treatment of malaria.

## 1. Introduction

Carbonic anhydrases (CAs; EC 4.2.1.1) compose a superfamily of metalloenzymes that owe the role of speeding up the carbon dioxide hydration to bicarbonate and proton^1^^,^^2^. Crucial biological processes in most organisms of tree of life are related to such a reversible reaction: respiration, photosynthesis, pH regulation, CO_2_ and HCO_3_^−^ transport, biosynthetic processes, production of body fluids, bone resorption, etc^3^^,^^4^. Eight evolutionarily unrelated CA classes have been identified to date, which are named as α-, β-, γ-, δ-, ζ-, η-, θ- and ι-CAs^4–8^. The α-CAs are present in vertebrates, protozoa, algae, corals, bacteria and cytoplasm of green plants^4^. Human, in particular, encode only for α-class isozymes^3^. The β-CAs have been identified in bacteria, fungi, Archaea, algae and chloroplasts of both mono- and dicotyledons^4^. The γ-CAs are encoded in Archaea, bacteria and plants^4^^,^^9^. δ-CAs have been discovered in marine phytoplankton, such as haptophytes, dinoflagellates, diatoms and chlorophytic prasinophytes, while ζ-CAs appear to be present only in marine diatoms^6^. A unique η-CA has been identified to date in the protozoa *Plasmodium falciparum*^7^. θ-CAs have been recently discovered in the marine diatom *Phaeodactylum tricornutum*^10^. A first specimen of ι-CAs was recently labelled from the marine diatom *Thalassiosira pseudonana*^8^.

**Figure 1.**
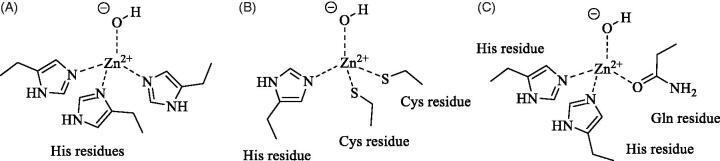
Metal ion coordination in the different CA families: (A) α-, γ - and δ-CAs (in the α- and δ-classes the coordinating residues are from the same monomer, whereas in γ -CAs the third His is from an adjacent monomer). (B) β-CAs (ζ -CAs possess a Cd(II) bound within the active site and show analogue coordination pattern). (C) η-CAs.

A unique δ-CA, TweCA, from the diatom *Thalassiosira weissflogii* was cloned and characterised in detail to date^11^, though orthologues of this enzyme have been identified in most diatoms from natural phytoplankton assemblages and are responsible (along with other CAs) for CO_2_ fixation by marine organisms^12^. TweCA is upregulated by low pCO_2_ and, under Zn-limited conditions, the zinc ion at the active site can be substituted by Co(II) *in vivo*^12^^,^^13^. TweCA is a protein of 281 amino acid residues. A subunit molecular mass of 32.0 kDa was estimated by SDS-PAGE, while the molecular mass of 32.2 kDa was calculated from the amino acid sequence. TweCA does not share any sequence homology to any other known CAs. The alignment of the amino acid sequence of TweCA with the polypeptide chain of the bovine α-CA (isoform bCA II) shows the low degree of identity with the mammalian α-CA^11^. Nonetheless, it was shown that the active site of TweCA is similar to that of mammalian α-CA^11^, with the metal coordination pattern formed by three histidines as found in α- and γ-CAs (Figure 1). Unfortunately, no structural data are available on δ-CAs. A phylogenetic analysis carried out using α-, γ- and δ-CAs from different prokaryotic and eukaryotic organisms showed that the α-CAs appear closely related to the δ-CAs, but clustered in a branch distinct from that of γ-CAs^14^. CA inhibitors, such as sulphonamides, inorganic anions, mono- and dithiocarbamates were screened as TweCA inhibitors^14–16^ with the aim to uncover molecular probes to investigate the role of this enzyme in the carbon fixation processes in marine diatoms that are responsible for removing large amounts of CO_2_ from the atmosphere.

The η-class of CAs was firstly described in 2015 by analysis of the amino acid sequences of CAs from *Plasmodia*, parasitic protozoa responsible of malaria in humans and other animals^7^. The first and unique member of the family to be characterised *in vitro* to date was *PfCA*, a protein of 600 amino acid residues, identified in *Plasmodium falciparum*^17–20^, one of the five species causing malaria in humans. Interestingly, *PfCA* was initially described as an α-CA enzyme, due to significant similarities with members of this class, but was subsequently reclassified into a new CA class, the η, due to some peculiar features^17^. In particular, the zinc coordination pattern of *PfCA* is formed by two histidines and one glutamine, distinctly from α-CAs, and many insertions and deletions in the protozoan enzyme were identified with respect to common α-CAs: insertions were observed in *PfCA* at the N-terminus and in the middle of the protein (69 additional residues after residue 152 of hCA II, chosen as reference protein). A three-dimensional model of *PfCA* was built by homology using the structure of *Thermovibrio ammonificans* CA (*Ta*CA) as template^21^. Because of low sequence homology only 267 residues (198–327 and 397–535) out of the 600 of the full-length protein could be modelled. A folding similar to that of α-CAs was found with the active site located in a large cavity with the zinc ion on the bottom (Figure 1), coordinated by His299, His301 and Gln320. The 69 residues insertion was located at the edge of the active site cleft, being presumably implicated in the catalysis^17^.

A significant interest is being dedicated to *PfCA*, because the enzyme has been recognised as possible target for the development of antimalarial drugs based on innovative mechanism of action. Indeed, a crucial role was suggested for *PfCA* in the *Plasmodium* parasites, being involved in the production of HCO_3_^−^ necessary as precursor of the pyrimidine biosynthetic pathway^22^. Its targeting to block this pathway could thus represent an efficient strategy for the development of new pharmacological agents against malaria^23^. In 1998, Sein and Aikawa showed that addition of CA inhibitors (CAIs) to a culture of *P. falciparum* provoked a remarkable reduction in parasitemia^24^. Successive reports illustrated that specific CA inhibition in *P. falciparum* and in the rodent parasite *P. berghei* produced the death of the parasite in *in vitro* cultures^22^. Starting from these data, the search of new *PfCA* inhibitors has started with sulphonamides and inorganic anions, and, though encouraging results have been obtained, more efforts are still necessary to obtain candidate drug molecules^18–20^.

Here, a series of phenolic derivatives (**1–22**, Figure 1) was assessed for the inhibition of TweCA and *PfCA* to extend such isoforms inhibition profiles, in search of novel leads for drug candidates or molecular probes which show the selective modulation of CAs from diatoms and protozoa over human isozymes.

## 2. Methods

### 2.1. Chemistry

Phenols **1–22** were commercially available from Sigma-Aldrich (Milan, Italy) and were used without further purification (purity >95%). All other reagents, salts, buffers and solvents were the highest purity available ones from Sigma-Aldrich (Milan, Italy).

### 2.2. Carbonic anhydrase inhibition

An Sx.18Mv-R Applied Photophysics (Oxford, UK) stopped-flow instrument has been used to assay the catalytic activity of various CA isozymes for CO2 hydration reaction^25^. Phenol red (at a concentration of 0.2 mM) was used as indicator, working at the absorbance maximum of 557 nm, with 20 mM TRIS (pH 8.3) as buffer, and 20 mM NaClO_4_ (for maintaining constant the ionic strength), following the initial rates of the CA-catalysed CO_2_ hydration reaction for a period of 10–100 s. The CO_2_ concentrations ranged from 1.7 to 17 mM for the determination of the kinetic parameters and inhibition constants. For each inhibitor, at least six traces of the initial 5–10% of the reaction have been used for determining the initial velocity. The uncatalyzed rates were determined in the same manner and subtracted from the total observed rates. Stock solutions of inhibitor (0.1 mM) were prepared in distilled-deionised water and dilutions up to 0.01 nM were done thereafter with the assay buffer. Inhibitor and enzyme solutions were preincubated together for 1 h at room temperature prior to assay, in order to allow for the formation of the E–I complex. The inhibition constants were obtained by nonlinear least-squares methods using PRISM 3 and the Cheng-Prusoff equation, as reported earlier, and represent the mean from at least three different determinations^26–28^. TweCAδ, *PfCA*, hCA I and II were recombinant proteins obtained in-house as reported earlier^29–31^.

## 3. Results and discussion

### 3.1. Selection of δ- and η-class CAs and chemistry

The kinetic parameters of the CO_2_ hydration reaction catalysed by TweCA and *PfCA* are reported in Table 1 in comparison with hCAs I and II. TweCA showed a significant catalytic activity with a k_cat_ of 1.3 × 10^5^ s^−1^ and a k_cat_/K_M_ of 3.3 × 10^7^ M^−1^ s^−1^. Similarly to β-, γ- and ζ-CAs, δ-CAs do not possess esterase activity.^14^ TweCA is stable up to 80 °C with residual activity of 40%, when the incubation time did not exceed 30 min. In contrast, bCA is inactivated at temperatures higher than 60 °C ^11^, suggesting that the δ-CA from *T. weissflogii* probably possess a more compact 3 D structure than other mammalian α-CAs.

**Table 1.** Kinetic parameters for the CO_2_ hydration reaction catalysed by the human cytosolic isozymes hCA I and II (α-class CAs), TweCAδ and *PfCA* measured at 20 °C^14^^,^^18^.

Enzyme	Species	Class	Activity level	K_cat_ (s^−1^)	k_cat_/k_m_ (M^−1^ × s^−1^)	K_I_ AAZ (nM)
hCA I	Human	α	Moderate	2.0 × 10^5^	5.0 × 10^7^	250
hCA II	Human	α	Very high	1.4 × 10^6^	1.5 × 10^8^	12
TweCAδ	*T. weissflogii*	δ	Moderate	1.3 × 10^5^	3.3 × 10^7^	83
*PfCA*	*P. falciparum*	η	Moderate	3.8 × 10^5^	7.2 × 10^7^	366

Data of Table 1 show that *PfCA* shows a significant catalytically activity for the CO_2_ hydration reaction, being the k_cat_ 3.8 × 10^5^ s^−1^ and the k_cat_/K_m_ of 7.2 × 10^7^ M^−1^ × s^−1^ ^18^. *PfCA* is more effective even compared to hCA I, and approximately 50% less effective compared to hCA II.

**Figure 2.**
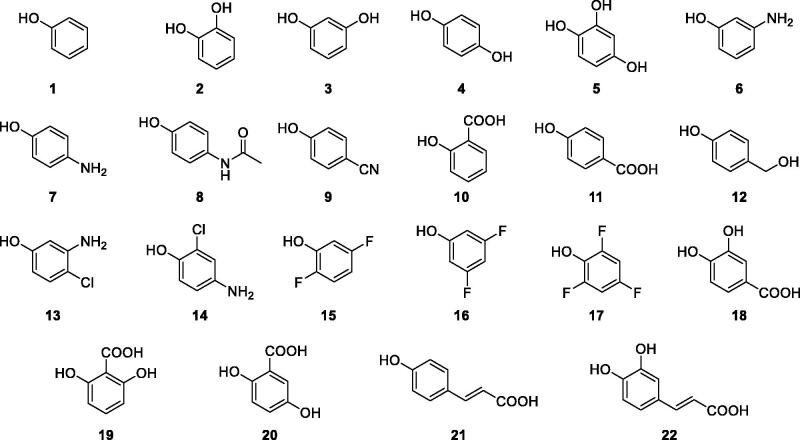
Structures of phenolic compounds **1–22**.

Phenolic compounds were shown to act as CAIs by a very distinct inhibition mechanism compared to primary sulphonamides, many of which are clinically used as diuretics, antiglaucoma, antiepileptic or in clinical trials for the management of advanced, hypoxic solid tumors^30^. In fact, whether sulphonamides directly coordinate the Zn(II) ion from the CA active site replacing the non-protein ligand, phenols were shown to anchor to the zinc-coordinated water molecule/hydroxide ion by a hydrogen bond network^30^. Up to now, phenolic derivatives, among which compounds **1–22** investigated here (Figure 2), were assayed as inhibitors of the human CA I, II, IX and XII^31^, of β-CAs, from the fungi *Saccharomyces cerevisiae, Candida albicans, Cryptococcus neoformans* and *Malassezia Globosa*^32^^,^^33^ or the bacterium *Mycobacterium tuberculosis*^34^ and γ-CAs from the pathogenic bacteria *Burkholderia pseudomallei*, *Pseudomonas gingivalis, Vibrio cholerae* and from the Antarctic bacteria *Pseudoalteromonas haloplanktis* and *Colwellia psychrerythraea*^35^.

As the δ- and η-CAs active sites are narrower than those of α-CAs, only phenyl derivatives, and not complex natural polyphenols, were considered^1^. A large variety of electron donating and electron withdrawing groups were investigated as substituents on the phenolic scaffold to uncover on the role of acidity of the anchoring group in the inhibitory activity (Table 2).

**Table 2.** Inhibition data of TweCAδ and *PfCA* with phenols **1–22** and the standard sulphonamide inhibitor acetazolamide (**AAZ**) by a stopped flow CO_2_ hydrase assay^27^.


Cmpd	R	K_I_ (µM)^a^			
		TweCAδ	*PfCA*	CA I^b^	CA II^b^
**1**	H	52.3	68.1	10.2	5.5
**2**	2-OH	4.5	1.4	>100	5.5
**3**	3-OH	48.2	26.9	>100	9.4
**4**	4-OH	34.9	21.0	10.7	0.1
**5**	2,4-diOH	2.0	0.83	>100	>100
**6**	3-NH_2_	65.4	78.7	4.9	4.7
**7**	4-NH_2_	17.6	33.8	>100	>100
**8**	4-NHCOCH_3_	7.9	26.4	10.0	6.2
**9**	4-CN	56.9	36.9	>100	0.1
**10**	2-COOH	0.95	0.72	9.9	7.1
**11**	4-COOH	1.4	0.90	9.8	10.6
**12**	4-CH_2_OH	35.9	47.1	68.9	95.3
**13**	3-NH_2_-4-Cl	>100	>100	6.3	4.9
**14**	4-NH_2_-2-Cl	>100	>100	57.8	57.5
**15**	2,5-diF	30.7	41.3	>100	>100
**16**	3,5-diF	21.0	32.7	38.8	33.9
**17**	2,4,6-triF	13.8	22.8	>100	>100
**18**	4-COOH-2-OH	4.9	1.6	1.1	0.5
**19**	2-COOH-3-OH	2.7	2.5	5.7	5.2
**20**	2-COOH-4-OH	0.81	0.62	4.2	4.1
**21**	4-(CHCHCOOH)	14.5	5.7	1.1	1.3
**22**	2-OH-4-(CHCHCOOH)	5.9	11.2	2.4	1.6
**AAZ**	–	0.08	0.36	0.25	0.01

^a^ Mean from 3 different assays, by a stopped flow technique (errors were in the range of ± 5–10% of the reported values)

^b^ Data from ref.^33^

### 3.2. δ- and η-class carbonic anhydrases inhibition

Phenols **1–22** were assayed as inhibitors of the unique δ- and η-class CAs identified to date, specifically from the marine diatom *T. weissflogii* and protozoan *P. falciparum*, respectively. A stopped flow CO_2_ hydrase assay was used including acetazolamide (**AAZ**) as standard inhibitor^25^. The inhibition profiles against the human ubiquitous CAs I and II are displayed for comparison^31^. The following structure–activity relationships (SAR) can be drawn up from the inhibition data reported in Table 2.

As a general trend, it can be stated that phenolic compounds are able to interfere with the CO_2_ hydrase activity of δ- and η-class CAs in the micromolar range. Inhibition constants (K_I_s) spanned, in fact, between 0.81 and 65.4 µM against TweCA and 0.62 and 78.7 µM against *PfCA*, while compounds **13** and **14** did not show inhibition below 100 µM.

It is fair to immediately stress that even carboxylic acids can act as CAIs, and can do that by two distinct mechanisms of action: coordination of the metal(II) ion or anchorage to the zinc-bound nucleophile. As a result, one cannot exclude that compounds **10**, **11**, **18–22**, which bear both phenolic and carboxylic groups, produce CA inhibition by the COOH function in place of the OH group.

Most substitutions at the phenol **1** scaffold produce enhancement in the inhibition of both TweCA and *Pf*CA, with the exception of *m*-substituents of the amine type (**6** and **13**) and an *o*-chlorine atom (**14**), that presumably induce significant steric hindrance for the binding in the active site. Also a *p*-CN group at the phenol scaffold led to light worsening of inhibitory action of **9** against TweCA in comparison to the lead **1** (K_I_s of 52.3 and 56.9 µM, respectively).

As for TweCA, a consistent subset of derivatives showed K_I_s lower than 10 µM (K_I_s in the range 0.81–7.9 µM). In particular, 1,2-diols **1** and **5** exhibited the most potent TweCA inhibition (K_I_s of 4.5 and 2.0 µM) among those compounds possessing solely OH and not COOH groups. On the other hand, swapping the second aromatic OH group to the *m*- or *p*- position did not produce a consistent increase of TweCA inhibition which settled for **3** and **4** at 48.2 and 34.9 µM. The substitution of hydrogens with fluorine atoms on the phenol scaffold increased the inhibition of TweCA by **15–17** (K_I_s in the range 13.8–30.7 µM) with respect to the lead **1**. In contrast, all benzoic derivatives reported low- to sub-micromolar activity. Precisely, the 2-hydroxy-benzoic acids **10** and **20** resulted to be the best TweCA inhibitors with submicromolar K_I_s of 0.95 and 0.81 µM. The presence of COOH group of the cinnamic acid type, such as in **21** or **22** did not elicit the same inhibition increased observed with benzoic acids, though the presence of a 1,2-diol portion in **22** drove its K_I_ against TweCA below 10 µM. None of the assayed compounds provoked as inhibitory effect as the reference **AAZ** (K_I_ of 83 nM).

As anticipated above, a superimposable inhibitory trend was measured for *Pf*CA with phenols **1–22** (Table 2). The 1,2,4-triol **5** showed inhibition of the plasmodial CA in the submicromolar range (K_I_ of 0.83 µM) reaching almost the same efficacy of benzoic acids **10**, **11**, **18–20** (K_I_s in the range 0.62–1.6 µM). Among the latter, the 2-hydroxybenzoic acid **20** stood out again as the best inhibitor here screened, also against *Pf*CA, with a K_I_ being less than the double of that shown by **AAZ** (K_I_ of 360 nM). The incorporation of a *p*-olefin portion in the 1,2-diol scaffold of **2** such as in **22** worsened the inhibitory efficacy from 1.4 to 11.2 µM. Swapping the second aromatic OH group to the *m*- or *p*- position produced a more consistent increase of *Pf*CA inhibition than that observed against TweCA, as the K_I_s of **3** and **4** settled at 26.9 and 21.0 µM. The parallelism observed in inhibition profile of TweCA and *Pf*CA with phenolic and/or carboxylic compounds might suggest similar binding modes of these chemotypes within the active sites of δ- and η-class CAs that, as stated above, both resemble that of α-CAs.

Table 3 reported the selectivity index (*SI*) calculated for TweCA and *Pf*CA over the human off-target CAs I and II. First, the inhibition profiles of hCAs I and II with phenols **1–22** should be briefly summarised. K_I_s against CA I show a peculiar trend as the half compounds are inhibitors in a low micromolar range below 10 µM, **12**, **14** and **16** exhibited K_I_s between 38.8 and 68.9 µM, whereas the remaining ones did not inhibit CA I below 100 µM. In contrast, a minor set of compounds did not inhibit hCA II (only **5**, **7**, **15** and **17**). Again **12**, **14** and **16** exhibited K_I_s above 10 µM (33.9–95.3 µM) and the most compounds effectively inhibited hCA II with K_I_s even reaching nanomolar such as in the cases of **4** and **9**. The *SI* values in Table 3 show that almost the half derivatives here screened exhibited selectivity of action against TweCA over hCAs I and II. The 1,2-diol **5** was the most selective among the screened compounds with *SI* over 50 against the diatom CA over off-target ones. Benzoic acids **10**, **11** and **20** also reported a significant selectivity of action with *SI* settling between 5 and 10 over both CA I and II. Also the trifluorophenol **17** displayed an interesting selectivity against TweCA over human CAs (*SI* of 7).

**Table 3.** Selectivity index (*SI*) for target CA over the off-target hCA I and II.

Cmpd	R	*SI*			
		I/TweCA	II/TweCA	I/*PfCA*	II/ *PfCA*
**1**	H	0.2	0.1	0.1	0.08
**2**	2-OH	>22.2	1.2	71.4	3.9
**3**	3-OH	>2.0	0.2	>3.7	0.3
**4**	4-OH	0.3	<0.01	0.5	<0.01
**5**	2,4-diOH	>50	>50	>100	>100
**6**	3-NH_2_	0.07	0.07	0.06	0.05
**7**	4-NH_2_	>5.6	>5.6	>2.9	>2.9
**8**	4-NHCOCH_3_	1.2	0.8	0.4	0.2
**9**	4-CN	>1.7	<0.01	>2.7	<0.01
**10**	2-COOH	10.4	7.4	13.7	9.8
**11**	4-COOH	7.0	7.5	10.9	11.8
**12**	4-CH_2_OH	1.9	2.6	1.4	2.0
**13**	3-NH_2_-4-Cl	<0.06	<0.05	<0.06	<0.05
**14**	4-NH_2_-2-Cl	<0.6	<0.5	<0.6	<0.6
**15**	2,5-diF	>3.2	>3.2	>2.4	>2.4
**16**	3,5-diF	1.8	1.6	1.1	1.0
**17**	2,4,6-triF	>7.2	>7.2	>4.3	>4.3
**18**	4-COOH-2-OH	0.2	0.1	0.6	0.3
**19**	2-COOH-3-OH	2.1	1.9	2.3	2.1
**20**	2-COOH-4-OH	5.2	5.0	6.8	6.2
**21**	4-(CHCHCOOH)	0.07	0.09	0.2	0.2
**22**	2-OH-4-(CHCHCOOH)	0.4	0.3	0.2	0.1
**AAZ**	–	3.1	0.1	0.7	0.03

Even higher *SI* were calculated against *Pf*CA over both hCAs I and II (Table 3). 1,2-Diols **2** and **5** showed the most selective and promising inhibition of the target *Pf*CA with respect to human CAs. While the *SI* of **2** settled at 70 and 4 over hCA I and II, respectively, those of **5** were even higher than 100 in both cases. The hCAs/*Pf*CA *SI* values were also increased with most benzoic acid derivatives with respect to those observed for TweCA. As sole exceptions, carboxylates **18**, **20** and **21** should be cited, since reported specificity of action for the human isozyme over the target ones (SI < 1). Analogue selectivity trend was observed for most other phenols (not showing COOH groups), such as **13** and **14**, which were particularly selective against hCAs over the plasmodial and diatom isozymes.

## 4. Conclusions

CAs of δ- and η-classes have not been extensively characterised from the inhibitory standpoint in comparison to α- and β-class isozymes. A unique δ-CA, TweCA, from the diatom *Thalassiosira weissflogii* was cloned and characterised in detail to date, though orthologues of this enzyme have been identified in most diatoms from natural phytoplankton assemblages and are responsible, along with other CAs for CO_2_ fixation by marine organisms. The identification of selective inhibitors of these isozymes is of significant importance to uncover molecular probes to investigate the role of this enzyme in the carbon fixation processes of marine diatoms that are responsible for removing large amounts of CO_2_ from the atmosphere.

Meanwhile a significant interest has been dedicated to *PfCA*, the unique specimen of η-CA, which was identified in *Plasmodium falciparum*, one of the five species causing malaria in humans. The research of *PfCA* inhibitors has started with sulphonamides and inorganic anions, and, though encouraging results have been obtained, more efforts are still necessary to obtain candidate drug molecules.

To extend TweCA and *PfCA* inhibition profiles, in search of novel leads for drug candidates or molecular probes selectively modulating these CAs over human isozymes, a panel of 22 phenols was investigated for these isozymes’ inhibition. The exploration of the chemical space around the main functional group led to the discovery of a number of such derivatives showing effective, sometimes sub-micromolar, inhibition against TweCA (K_I_s 0.81 and 65.4 µM) and *PfCA* (K_I_s 0.62 and 78.7 µM). A subset of compounds even demonstrated a significant selectivity for the target CAs over the human physiologically relevant isoforms CA I and II. This study improves the knowledge on the modulation of CAs belonging to uncommon classes such as δ and η. As a result, it promotes the identification of new potent and selective inhibitors against diatom and plasmodial isoforms over human off-target CAs, which could be adopted as leads for finding molecular probes in the study of carbon fixation processes or drug candidates in the treatment of malaria.
